# ATN profile classification across two independent prospective cohorts

**DOI:** 10.3389/fmed.2023.1168470

**Published:** 2023-07-25

**Authors:** Débora E. Peretti, Federica Ribaldi, Max Scheffler, Linjing Mu, Valerie Treyer, Anton F. Gietl, Christoph Hock, Giovanni B. Frisoni, Valentina Garibotto

**Affiliations:** ^1^Laboratory of Neuroimaging and Innovative Molecular Tracers (NIMTlab), Faculty of Medicine, Geneva University Neurocenter, University of Geneva, Geneva, Switzerland; ^2^Laboratory of Neuroimaging of Aging (LANVIE), University of Geneva, Geneva, Switzerland; ^3^Division of Radiology, Geneva University Hospitals, Geneva, Switzerland; ^4^Department of Nuclear Medicine, University Hospital of Zurich, University of Zurich, Zurich, Switzerland; ^5^Institute of Pharmaceutical Sciences, Zurich, Switzerland; ^6^Institute for Regenerative Medicine (IREM), University of Zurich, Zurich, Switzerland; ^7^Memory Clinic, Geneva University Hospitals, Geneva, Switzerland; ^8^Division of Nuclear Medicine and Molecular Imaging, Geneva University Hospitals, Geneva, Switzerland; ^9^Center for Biomedical Imaging, University of Geneva, Geneva, Switzerland

**Keywords:** ATN profiles, positron emission tomography, biomarkers, cognition, classification

## Abstract

**Background:**

The ATN model represents a research framework used to describe in subjects the presence or absence of Alzheimer’s disease (AD) pathology through biomarkers. The aim of this study was to describe the prevalence of different ATN profiles using quantitative imaging biomarkers in two independent cohorts, and to evaluate the pertinence of ATN biomarkers to identify comparable populations across independent cohorts.

**Methods:**

A total of 172 subjects from the Geneva Memory Clinic and 113 volunteers from a study on healthy aging at the University Hospital of Zurich underwent amyloid (A) and tau (T) PET, as well as T1-weigthed MRI scans using site-specific protocols. Subjects were classified by cognition (cognitively unimpaired, CU, or impaired, CI) based on clinical assessment by experts. Amyloid data converted into the standardized centiloid scale, tau PET data normalized to cerebellar uptake, and hippocampal volume expressed as a ratio over total intracranial volume ratio were considered as biomarkers for A, T, and neurodegeneration (N), respectively. Positivity for each biomarker was defined based on previously published thresholds. Subjects were then classified according to the ATN model. Differences among profiles were tested using Kruskal-Wallis ANOVA, and between cohorts using Wilcoxon tests.

**Results:**

Twenty-nine percent of subjects from the Geneva cohorts were classified with a normal (A−T−N−) profile, while the Zurich cohort included 64% of subjects in the same category. Meanwhile, 63% of the Geneva and 16% of the Zurich cohort were classified within the AD continuum (being A+ regardless of other biomarkers’ statuses). Within cohorts, ATN profiles were significantly different for age and mini-mental state examination scores, but not for years of education. Age was not significantly different between cohorts. In general, imaging A and T biomarkers were significantly different between cohorts, but they were no longer significantly different when stratifying the cohorts by ATN profile. N was not significantly different between cohorts.

**Conclusion:**

Stratifying subjects into ATN profiles provides comparable groups of subjects even when individual recruitment followed different criteria.

## 1. Introduction

Alzheimer’s disease (AD) is pathologically defined by the deposition of extracellular amyloid (A) plaques and intracellular neurofibrillary tau (T) tangles (1). In addition to the A and T biomarkers, a third biomarker based on neurodegeneration (N) is also used, although N has been considered more for disease staging than for disease definition (2). These biomarkers can be expressed in a binary form: subjects are considered either positive or negative for A, T, and N separately. Then, an ATN profile can be constituted for each individual. According to the model of Jack et al. (3), A+ is sufficient to be labeled as Alzheimer’s continuum, A + T+ (N+ or N−) defines Alzheimer’s pathologic change, while A−T+ or A−N+ are labeled as non-Alzheimer’s pathologic change. The ATN model was first introduced so that a biological definition of AD could be used in research frameworks, instead of relying on clinical presentations of the disease (3). Even if the classification provides a simplified description of a subject’s pathology, it has been shown to be able to predict the risk for clinical progression to dementia (4). With this, the ATN classification has the potential to refine inclusion criteria for AD clinical trials and drug assessment.

A, T, and N biomarkers can be measured through positron emission tomography (PET) quantification, magnetic resonance imaging (MRI), cerebral spinal fluid (CSF), or peripheral blood-based biomarkers (5). However, these approaches cannot be used interchangeably, and each has their advantages and drawbacks. CSF- and PET-based biomarkers are the reference standard in the identification of patients in the AD continuum (3). A and T biomarkers measured through these techniques allow to detect subjects more likely to develop AD dementia (6–8), while N is associated with higher dementia risk, but is not a specific biomarker for AD (6, 9). However, CSF and PET measure different forms of amyloid and tau, which might increase heterogeneity in trial populations (10). In fact, T measured through PET offers a more accurate prognosis at the time of subject recruitment when compared to CSF measures (11, 12), along with its advantage of portraying the topographic distribution of the tau tangles across the brain, which allows for subject Braak staging (13). Finally, the blood-based approach to measure biomarkers is the least invasive and has the lowest cost. While the use of these biomarkers could provide an easily accessible approach to assess AD pathology, considerable work remains to be done before this measure could be implemented as a clinical test (14–16). Furthermore, some phosphorylated tau assays have been shown to have a better correlation with A than with T (17), questioning the pathology it describes (18).

The identification of ATN profiles in research and clinical populations has gained great attention in the past years (4, 19–28). Most of the studies included a combination of PET imaging and CSF for the assessment of different biomarkers. Very recently, Dodich et al. (21), analyzed ATN biomarkers by PET and/or MRI techniques, which could provide precise and consistent characterization of ATN profiles (29, 30).

The aim of this study was to describe the prevalence of ATN profiles using quantified imaging biomarkers, namely PET and MRI, alone in two independent samples of subjects using center-specific protocols. Furthermore, it also aimed to evaluate the pertinence of ATN biomarkers to identify comparable populations across independent cohorts.

## 2. Materials and methods

### 2.1. Geneva cohort

A prospective cohort of 172 subjects was included from the Geneva Memory Clinic at the Geneva University Hospitals (HUG), Geneva, Switzerland. The local review board (Commission cantonale d’éthique de la recherche—CCER de Genève) approved the study, which was conducted in concordance with the principles of the Declaration of Helsinki and the International Conference on Harmonization Good Clinical Practice. Each subject provided written informed consent for participation in the study. Subjects underwent clinical, neurological, and neuropsychological assessment (including mini-mental state examination—MMSE), MRI, and amyloid and tau PET scans. Subjects were diagnosed with a variety of diagnoses: healthy control individuals (HC), and patients with subjective cognitive decline (SCD), mild cognitive impairment (MCI), or dementia. Diagnosis was based on a clinical assessment combined with the results of the neuropsychological assessment. Subjects with SCD were evaluated with a self-experience of deterioration in cognitive abilities but did not present objective cognitive impairment through formal neuropsychological testing (31). MCI patients presented objective cognitive impairment and no functional impairing in everyday life (32). Individuals were diagnosed with dementia if they matched MCI requirement but differ from MCI subjects for the impairment in everyday life (33). Included subjects were then classified by cognition status: cognitively unimpaired (CU: HC and SCD subjects) or cognitively impaired (CI: MCI and dementia patients).

PET scanning was performed at the Nuclear Medicine and Molecular Imaging Division at HUG. For amyloid imaging, 73 subjects were injected with 194 ± 29 MBq of [^18^F]florbetapir, and images were acquired 50 min after intravenous administration of the radiotracer (3 × 5 min image frames that were averaged into a single image). The remaining 99 subjects were scanned with 185 ± 27 MBq of [^18^F]flutemetamol, and images were acquired 90 min after intravenous administration of the radiotracer (4 × 5 min image frames that were averaged into a single image). All subjects underwent tau PET imaging using 202 ± 46 MBq of [^18^F]Flortaucipir and was acquired 75 min after tracer injection (6 × 5 min image frames that were averaged into a single image). All images were acquired using a Siemens Biograph PET scanner (Siemens Healthineers, Germany), reconstructed using 3D OSEM algorithm (4 iterations 8 subsets) and a 2 mm Gaussian convolution kernel, corrected for dead time, normalization, attenuation, and sensitivity. All radiotracers were approved by the US Food and Drug Administration and the European Medicine Agency for clinical PET studies in patients. All radiotracers are commercially available and were synthesized at radiopharmaceutical Good Manufacturing Practice laboratories and shipped to Geneva.

All subjects underwent MRI scans at the Radiology Division at the HUG using a 3 Tesla Siemens Magneton Skyra scanner (Siemens Healthineers, Germany) equipped with a 64-channel head coil. 3D T1-weighted images were acquired with a square field of view of 256 mm, 0.9 mm slice thickness, 1930 ms repetition time, 2.4 ms time to echo, 8° flip angle, and no fat suppression. Images were acquired corresponding to IMI Pharmacog WP5/European ADNI sequences and published procedures (34). All imaging modalities were performed within a 1-year period of each other.

### 2.2. Zurich cohort

A cohort of 113 subjects was included form a prospective study in healthy aging at the University Hospital Zurich (USZ), Zurich, Switzerland. The local review board approved the study, which was conducted un concordance with the principles of the Declaration of Helsinki and the International Conference on Harmonization Good Clinical Practice. Each subject provided written informed consent for participation in the study. Subjects underwent neurological evaluation, magnetic resonance imaging, and amyloid and tau PET scans. Included subjects were diagnosed either as HC or MCI. For the HC subjects, inclusion criteria were age between 50 and 80 years, unimpaired overall cognitive status as indicated by an MMSE score of 27 or above, neuropsychological testing, and comprehensive psychiatric examination. Participants were determined to be cognitively unimpaired or fulfilling criteria for MCI as determined by a diagnostic conference that included at least one experienced clinician and one neuropsychologist, incorporating all available clinical information and according to published diagnostic guideline (35). Exclusion criteria were the presence of any condition possibly affecting cognition, any current medication or substance abuse with prompt effects on cognition, serious medical or psychiatric illness, and evidence of infarction or inflammation on cranial MRI. To match the Geneva cohort, subjects were separated into CU (HC subjects) and CI (MCI patients) groups as well.

PET scanning was performed at the Department of Nuclear Medicine at the USZ. For amyloid imaging, 75 subjects were injected with an average of 140 MBq of [^18^F]flutemetamol. Subjects received a standard dynamic PET scan using a Signa PET/MR (GE HealthCare, United States). Images were averaged between 85 and 105 min after tracer injection (4 × 5 min frames that were averaged into a single image). The remaining 40 subjects were injected with an average of 350 MBq of ^11^C-labeled Pittsburgh compound B. Subjects received a standard dynamic PET scan using a Discovery PET/CT (GE HealthCare, United States). Image frames (4 × 5 min frames) between 50 and 70 min after tracer injection were averaged into a single image. For tau imaging, all subjects underwent a second [^18^F]flortaucipir PET scan, with an average injection of 200 MBq. Subjects underwent a standard dynamic PET scan using a Sigma PET/MR (GE HealthCare, United States). Image frames (4 × 5 min frames) between 80 and 100 min after tracer injection were averaged into a single image. [^18^F]flutemetamol and [^18^F]flortaucipir acquisitions were reconstructed into MRAC images to derive attenuation correlation maps according to standard manufacturer implemented algorithms with time-of-flight 3D OSEM reconstruction. Meanwhile, ^11^C-labeled Pittsburgh compound B acquisitions were reconstructed according to standards with 3D OSEM filtered back projection, CT attenuation correction, scatter, randoms, and deadtime as well as sensitivity corrections. All radiotracers were approved by the US Food and Drug Administration and the European Medicine Agency for clinical PET studies in patients. All radiotracers are commercially available and were synthesized at radiopharmaceutical Good Manufacturing Practice laboratories at the Swiss Federal Institute of Technology in Zurich or at the USZ. MRI was performed using a BRAVO 3D T1 MRI sequence with a voxel size of 1 mm (8-channel coil).

### 2.3. Image processing

Images were thus acquired in each center separately with center-specific protocols. However, all images from both cohorts were processed at the Geneva Memory Centre at the HUG, using SPM12 (Wellcome Trust Centre for Neuroimaging, London, United Kingdom) and MATLAB R2018b version 9.5 (MathWorks Inc., Sherborn, United States). First, 3D T1-weighted MRI images were aligned to the anterior commissure—posterior commissure line. Then, they were normalized to the Montreal Neurologic Institute (MNI) space using tissue probability maps (36). PET images were aligned to the subject’s respective MRI images and then, using the MRI transformation matrix, they were transformed into the MNI space.

For the tau PET images, standardized uptake value rations (SUVR) were generated using the cerebellum as a reference region (13, 37), and data were extracted using the automated anatomic labeling atlas 3 (38, 39). To be able to compare between the different A radiotracers, amyloid PET images were converted into the centiloid scale following the standard centiloid processing pipeline (40), the only adaptation being the use of SPM12 instead of SPM8, which has been previously shown to provide comparable extracted values (41). Conversion from SUVR to centiloid scale was performed using previously published equations that were fully validated (40, 42, 43). Cortical reconstruction and volumetric segmentation of T1 images were performed using Freesurfer [v7, recon-all (44)]. Right and left hippocampal volumes were extracted separately, averaged, and normalized to the total intracranial volume.

### 2.4. ATN classification

Each imaging biomarkers was considered individually, and their positivity was decided based on previously published thresholds. Subjects were considered amyloid positive (A+) if centiloid value was above 12 (45). The choice of a low centiloid threshold was done to detect subjects at an early stage of accumulation (46).

Tau positivity was considered based on the Simplified Temporal-Occipital Classification (STOC) (13). In summary, four bilateral brain regions were considered: medial temporal lobe (MTL), lateral temporal lobe (LTL), superior temporal gyrus (STG), and primary visual cortex (PVC). If the SUVR value from a region was above an established threshold (1.24 for MTL, 1.31 for LTL, 1.26 for STG, and 1.31 for PVC), that region was considered positive for tau accumulation. STOC stage was defined based on regional positivity. If no regions were positive, the subject was classified as stage 0. Stages 1 to 4 were defined depending on which regions were positive: MTL alone, stage 1; MTL and LTL, or LTL alone (hippocampal sparing), stage 2; MTL, LTL, and STG, or LTL and STG (hippocampal sparing), stage 3; MTL, LTL STG, and PVC, or LTL, STG, and PVC (hippocampal sparing), stage 4. Subjects with any other combination of positive regions were considered stage “Atypical.” Subjects in stages 0 or 1 were considered negative for tau accumulation (T−), and subjects in stages 2, 3, 4, or Atypical were considered positive (T+). Atypical subjects were considered T+ based on their global SUVR values and a 1.24 threshold previously estimated using the Geneva cohort (21). To assess differences between profiles, tau uptake was summarized in a composite bilateral global SUVR value including amygdala, parahippocampus, middle occipital gyrus, and inferior temporal gyrus (47).

Neurodegeneration positivity (N+) was decided when subject’s ratio of hippocampal volume to total intracranial volume (“Hippocampal Ratio”) was below 0.00215 (48). This threshold was internally validated against other imaging approaches and is adequate for the assessment of N positivity for images acquired using the systems previously described (49).

### 2.5. Statistical analysis

To compare between CU and CI groups, a Mann–Whitney U test was used to explore differences in age, MMSE, centiloid values, global tau SUVR, and hippocampal ratio for each cohort separately. Then, the same test was used to compare differences in the same parameters between cohorts for CU and CI groups separately. A chi-square test was used to compare ATN distributions by cognition group between cohorts and to test if the distribution of subjects within the typical amyloid cascade theory profiles for AD was different between cohorts. To assess similarities and differences between cohorts, for each ATN profile, a Mann–Whitney U test was used to compare centiloid values, global tau SUVR, and hippocampal ratio between cohorts. Differences among profiles were tested using Kruskal-Wallis ANOVA for each cohort separately, with correction for multiple comparisons using the Dunn test. An ANOVA test to predict MMSE values using cohort, ATN profile, and the interaction between both variables was used to test the effect of these variables on global cognition.

A *p*-value of 0.05 was considered as significance threshold for all analyses, which were performed using RStudio (version 2022.07.1, R version 4.2.1). Comparisons between groups of less than 5 individuals were disregarded as there was not enough power to consider results statistically sound.

## 3. Results

### 3.1. Population

Table 1 shows demographic, cognitive, and imaging characteristics of both cohorts included in this study. While the Geneva cohort showed no significant difference between CU and CI for age, the Zurich cohort did. Meanwhile, the Zurich cohort showed no significant differences between groups for years of education and centiloid values, while the Geneva cohort did. Both cohorts showed significant differences between CU and CI subjects in MMSE scores, global tau SUVR uptake, and hippocampal ratio. Both cohort and ATN status were significant predictors of MMSE scores, but the interaction between these variables was not. Supplementary Table S1 shows demographic and imaging characteristics of both cohorts included in this study by ATN profile.

**Table 1:** Descriptive features of the included cohorts.

	Cohort	CU	CI	*p*-value
Number of subjects	Geneva	42	130	–
	Zurich	82	31	–
Age (y)	Geneva	72 ± 7	73 ± 8	0.27
	Zurich	70 ± 9	75 ± 8	<0.01
	*p*-value	0.15	0.14	–
Gender (F/M)	Geneva	25/17	61/69	0.15
	Zurich	31/51	8/23	0.23
	*p*-value	0.02	0.03	–
Years of education (y)	Geneva	16 ± 4	13 ± 4	<0.01
	Zurich	16 ± 3	16 ± 3	0.73
	*p*-value	0.37	<0.01	–
MMSE	Geneva	28 ± 1	25 ± 4	<0.01
	Zurich	29 ± 1	28 ± 2	<0.01
	*p*-value	< 0.01	<0.01	–
Centiloid (A)	Geneva	11 ± 33	66 ± 47	<0.01
	Zurich	3 ± 10	11 ± 27	0.38
	*p*-value	0.89	<0.01	–
Global tau SUVR (T)	Geneva	1.15 ± 0.13	1.41 ± 0.32	<0.01
	Zurich	1.10 ± 0.13	1.17 ± 0.14	<0.01
	*p*-value	0.09	<0.01	–
Hippocampal ratio* (×10^−3^) (N)	Geneva	2.5 ± 0.2	2.3 ± 0.3	<0.01
	Zurich	2.4 ± 0.3	2.3 ± 0.3	<0.01
	*p*-value	0.26	0.84	–

Demographic, cognitive, and imaging characteristics of subjects in the study by cognitive status and cohort. Reported *p*-values as derived from Wilcoxon tests (within and between cohorts), and from the Chi-squared test for the gender variable (within and between cohorts). CU, cognitively unimpaired; CI, cognitively impaired; y, years; F, female; M, male; MMSE, mini-mental state examination; A, amyloid; T, tau; N, neurodegeneration; SUVR, standardized uptake value ratio. *Ratio between hippocampal volume and total intracranial volume.

**Figure 1:**
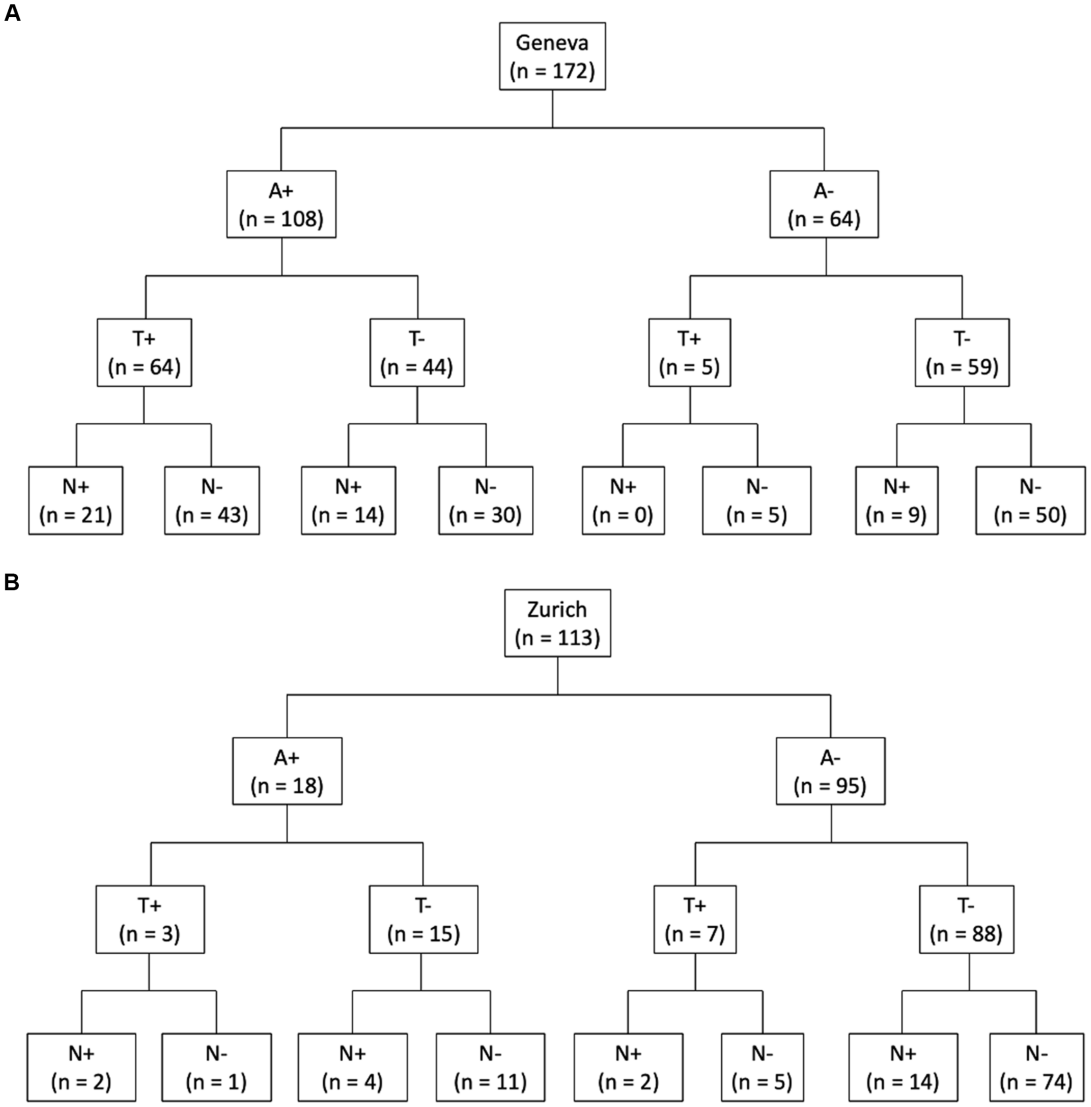
ATN classification. Flow chart of ATN classification of subjects with amyloid and tau PET and T1 MRI scans for Geneva **(A)** and Zurich **(B)** cohorts. A, amyloid; T, tau; N, neurodegeneration; n, number of subjects.

**Figure 2:**
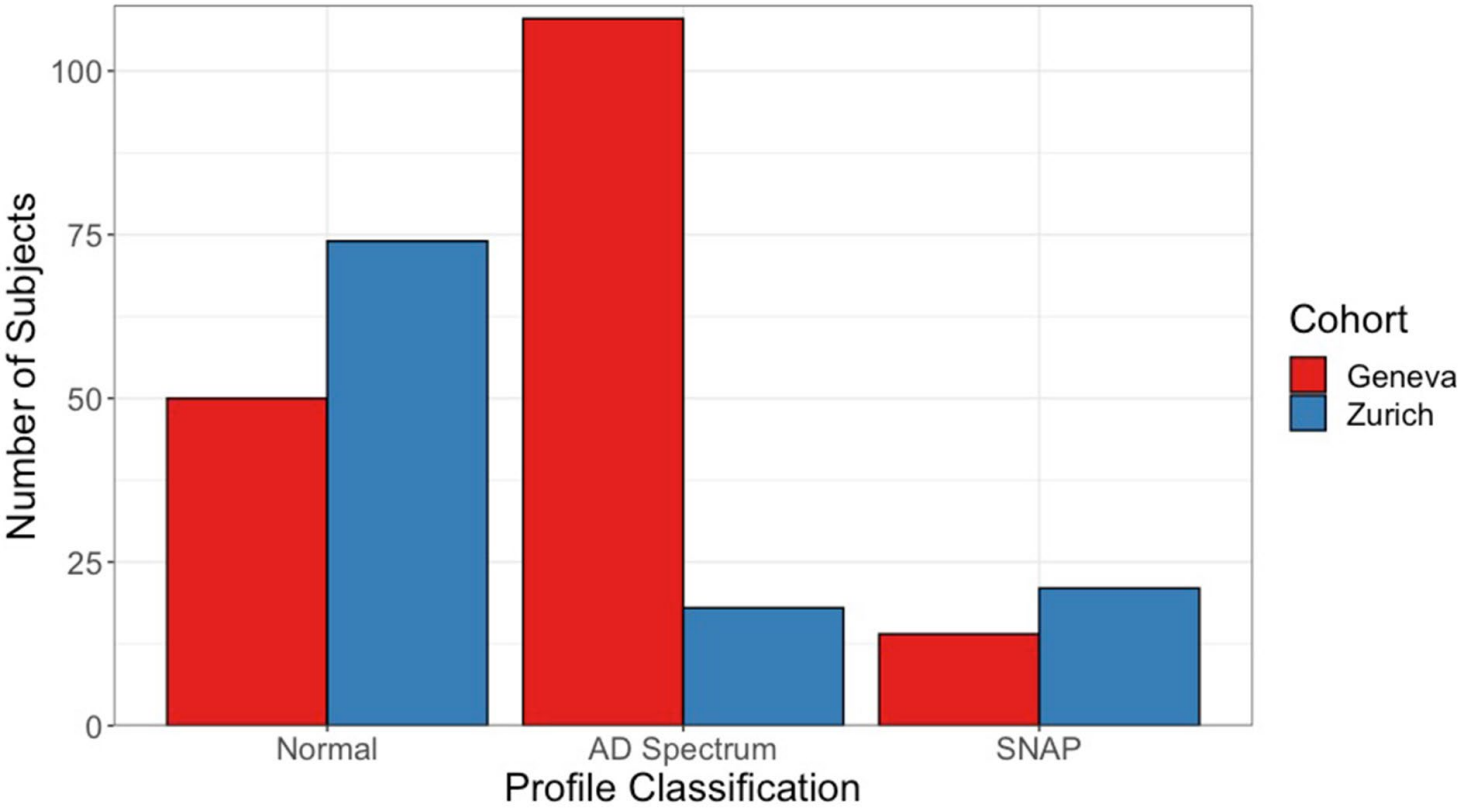
Frequency of ATN profile classification by cohort. Number of subjects in each classification for ATN profiles. Normal classification comprises subjects classified with an A−T−N− profile. Subjects within the AD continuum classification present a profile with A+ status (A + T−N−, A + T + N−, A + T + N+, A + T−N+). Finally, SNAP classification includes A−T + N−, A−T−N+, and A−T + N+ profiles. Red (left) bars of bar pair show subjects from the Geneva cohort, and blue (right) bars from the Zurich cohort. AD, Alzheimer’s disease; SNAP, suspected non-Alzheimer’s disease pathophysiology.

Figure 1 shows the flowchart for ATN profile classification and number of subjects per profile for both cohorts. Furthermore, Figure 2 shows the number of subjects in different profile classifications. The normal classification includes subjects with negative biomarkers (A−T−N− profile). Subjects with A+ profiles (A + T−N−, A + T + N−, A + T + N+, and A + T−N+) were classified part of the AD continuum. Remaining profiles (A−T + N−, A−T−N+, and A−T + N+) were classified as suspected non-AD disease pathophysiology (SNAP). In the Geneva cohort, the most prevalent profiles were profiles within the AD spectrum (62.7%), follow by the normal profile (29.1%), and SNAP (8.2%). Meanwhile, in the Zurich cohort, the normal profile was the most prevalent (65.5%), followed by SNAP (18.6%), and AD continuum (15.9%).

### 3.2. Centiloid and amyloid status

**Figure 3:**
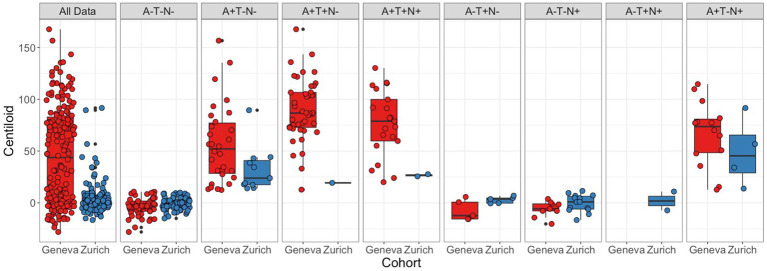
Centiloid distribution by ATN status. Centiloid value distribution for each cohort per diagnosis. Red boxplot shows data from the Geneva cohort, and blue boxplots the Zurich cohort. Panels with subjects’ ATN status from left to right: complete data, A−T−N−, A + T−N−, A + T + N−, A + T + N+, A−T + N−, A−T−N+, A−T + N+, A + T−N+. Boxes represent the interquartile range of score distribution; the horizontal lines indicate the median score per diagnosis; whiskers expand up to 1.5 times the interquartile ranges; and the remaining black dots correspond to outliers. Colored circles represent individual values.

Average and standard deviation of centiloid values per cognitive status and cohort are described in Table 1. Results in this table show significant differences in cognitive statuses within and between cohorts. The distribution of centiloid values for the complete data and per ATN profile and cohort is shown in Figure 3. Table 2 shows mean and standard deviation of centiloid values per ATN profile for both cohorts.

**Table 2:** Imaging values of the cohorts by ATN profile.

Profile	n (CU/CI)		Centiloid		Global tau SUVR		Hippocampal ratio* (×10^−3^)	
	Geneva	Zurich	Geneva	Zurich	Geneva	Zurich	Geneva	Zurich
A−T−N−	50 (27/23)	74 (60/14)	−4 ± 9	−1 ± 6	1.1 ± 0.1	1.1 ± 0.1	2.6 ± 0.2	2.5 ± 0.2
A + T−N−	30 (9/21)	11 (9/2)	55 ± 38	32 ± 22	1.2 ± 0.1	1.1 ± 0.1	2.5 ± 0.2	2.4 ± 0.2
A + T + N−	43 (1/42)	1 (1/0)	90 ± 30	19 ± 0	1.7 ± 0.3	1.8 ± 0.0	2.4 ± 0.2	2.2 ± 0.0
A + T + N+	21 (0/21)	2 (0/2)	77 ± 32	27 ± 1	1.6 ± 0.2	1.3 ± 0.0	1.9 ± 0.2	2.1 ± 0.1
A−T + N−	5 (4/1)	5 (3/2)	−7 ± 10	3 ± 3	1.3 ± 0.1	1.4 ± 0.1	2.5 ± 0.2	2.4 ± 0.2
A−T−N+	9 (1/8)	14 (8/6)	−6 ± 8	0 ± 8	1.1 ± 0.1	1.1 ± 0.1	2.0 ± 0.1	1.0 ± 0.2
A−T + N+	0 (0/0)	2 (1/1)	–	2 ± 13	–	1.3 ± 0.1	–	2.0 ± 0.2
A + T−N+	14 (0/14)	4 (1/3)	67 ± 31	49 ± 33	1.2 ± 0.1	1.2 ± 0.1	1.9 ± 0.3	2.0 ± 0.1

Centiloid, global tau SUVR, and hippocampal ratio average and standard deviations by ATN profile for the Geneva and Zurich cohorts. A, amyloid; T, tau; N, neurodegeneration; n, number of subjects; CU, cognitively unimpaired; CI, cognitively impaired. *Ratio between hippocampal volume and total intracranial volume.

In the Geneva cohort, 63% of subjects were classified as A+ (*n* = 108), while in the Zurich cohort, only 16% of subjects were classified as A+. When comparing centiloid values between cohorts in general, significant differences between cohorts were found. However, when comparing between cohorts by ATN profile, no significant differences were found. Centiloid values were significantly different between profiles for both cohorts. In the Geneva cohort, the A−T−N−, A−T + N−, and A−T−N+ groups were not significantly different from each other but were significantly lower than A + T−N−, A + T + N−, A + T + N+, and A + T−N+ profiles. Furthermore, in Geneva, centiloid values in the A + T−N− profile were significantly lower than in the A + T + N− profile. In the Zurich cohort, the A−T−N− and A−T−N+ profiles were not significantly different from each other but had significantly lower centiloid values than the A + T−N− profile. Furthermore, the A−T−N− and A−T−N+ profiles were not significantly different from each other but had significantly lower centiloid values than the A + T−N+ profile.

### 3.3. Global tau SUVR and tau status

**Figure 4:**
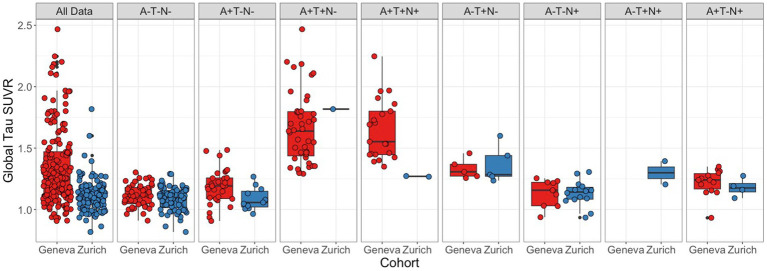
Global tau SUVR distribution by ATN status. Distribution of subjects’ global tau SUVR uptake by cohort for all subjects and by ATN status, from left to right: A−T−N−, A + T−N−, A + T + N−, A + T + N+, A−T + N−, A−T−N+, A−T + N+, A + T−N+. Boxes represent the interquartile range of score distribution; horizontal lines the median scores per diagnosis; whiskers expand up to 1.5 times the interquartile range; and the remaining black dots correspond to outliers. Colored circles represent individual values.

Table 1 shows the average and standard deviation of global tau SUVR values per cognitive status and significant differences in cognitive statuses within and between cohorts. Figure 4 displays the distribution of values of the complete data for each cohort of the complete data and per ATN profile. Table 2 shows average and standard deviation global tau SUVR by ATN profile for both cohorts.

In the Geneva cohort, 40% of subjects were classified as T+ (*n* = 69), while only 8% (*n* = 10) of subjects in the Zurich cohort were classified as T+. In both cohorts most of the T− subjects were also classified as A−. While the Geneva cohort the majority of T+ cases was classified within the A+ classification, T+ subjects from the Zurich cohort were mostly within the A− classification. When comparing global tau SUVR between cohorts, a significant difference was found. However, as in the amyloid data, when stratifying subjects into ATN profiles, the differences between cohorts were no longer significant. In general, ATN profiles had significantly different global tau SUVR values in both cohorts. When correcting for multiple comparisons, in the Geneva cohort, the A−T−N− profile had significantly lower global tau SUVR values than the A−T + N−, A + T + N−, and A + T + N+ profiles; the A + T−N− profile had significantly lower tau SUVR values than the A + T + N− and A + T + N+ profiles; the A + T−N+ profile had significantly lower tau SUVR when compared to the A + T + N− and A + T + N+ profiles; and the A−T−N+ profile had significantly lower tau SUVR values than the A + T + N− and A + T + N+ profiles. Meanwhile, in the Zurich cohort, the A−T + N− had significantly higher tau SUVR values when compared to the A−T−N−, and A + T−N− profiles.

### 3.4. Adjusted hippocampal volume and neurodegeneration status

**Figure 5:**
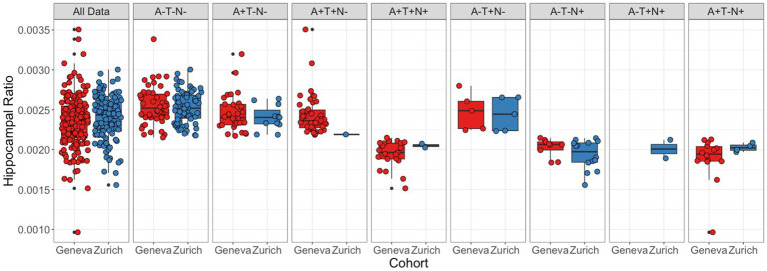
Distribution of Adjusted Hippocampal Volume Values by ATN status. Distribution of subjects’ hippocampal ratio by cohort for all subjects and by ATN status, from left to right: A−T−N−, A + T−N−, A + T + N−, A + T + N+, A−T + N−, A−T−N+, A−T + N+, A + T−N+. Boxes represent the interquartile range of value distribution; horizontal lines the median score; the whiskers expand up to 1.5 times the interquartile ranges; remaining black dots correspond to outliers. Colored circles represent individual values.

Average and standard deviation of hippocampal ratio per cognitive status and cohort are described in Table 1. Differences between and within cohorts are also shown in Table 1. Distribution of values for the complete data and per ATN status for both cohorts are shown in Figure 5, and average and standard deviations, in Table 2.

In the Geneva cohort, 26% of subjects were classified as N+ (*n* = 44), while 19% (*n* = 22) of subjects in the Zurich cohort had the same classification. Most of the N+ subjects in the Geneva cohort were within the A + T+ group, while most of the N+ subjects in the Zurich cohort had an A−T− classification. Differences between cohorts were not significantly different neither when considering the complete data, nor when considering ATN profiles individually. In general, hippocampal ratio values were significantly different between profiles in both cohorts. In the Geneva cohort, when analyzing the pairs and correcting for multiple comparisons, the A−T−N− profile was significantly larger than the A−T−N+, A + T−N+, A + T + N−, and A + T + N+ profiles; the A−T + N− was larger than the A + T−N+, A + T + N+, and A−T−N+ profiles; the A−T−N+ was smaller than the A + T−N−, and A + T + N− profiles; the A + T−N− was larger than the A + T−N+ and A + T + N+ profiles; and the A + T + N− was larger than the A + T−N+ and A + T + N+ profiles. In the Zurich cohort, the A−T−N− profile had significantly larger hippocampal ratio values than the A−T−N+ and A + T−N+ profiles; and the A−T−N+ has significantly smaller hippocampal ratio values than the A−T + N− and A + T−N− profiles.

### 3.5. ATN distribution

Table 2 shows the frequency of subjects in each ATN profile for both cohorts. Both cohorts had the A−T−N− profile as the most frequent one, with 50 subjects (29%) in the Geneva cohort and 74 (65%) in the Zurich cohort. In the Geneva cohort, the second most frequent profile was A + T + N− (*n* = 43), followed by A + T−N− (*n* = 30), A + T + N+ (*n* = 21), A + T−N+ (*n* = 14), A−T−N+ (*n* = 9), and A−T + N− (*n* = 5). Meanwhile, in the Zurich cohort, the second most frequent profile was A−T−N+ (*n* = 14), followed by A + T−N− (*n* = 11), A−T + N− (*n* = 5), A + T−N+ (*n* = 4), A + T + N+ (*n* = 2), A−T + N+ (*n* = 2), and A + T + N− (*n* = 1). A significant difference was found when comparing ATN profile distribution between cohorts (*p* < 0.01). Supplementary Table S2 shows the same data from Table 2 but also separated by cognitive status.

When comparing MMSE scores between cohorts for each ATN profile, three profiles presented a significant difference: A−T−N− (*p* < 0.01), A + T−N− (*p* < 0.01), A + T + N+ (*p* < 0.01), and A−T−N+ (*p* < 0.01). The A−T + N− profile was not significantly different between cohorts. ATN profiles A + T + N−, A + T + N+, A−T + N+, and A + T−N+ could not be compared due to the small number of subjects with each of these profiles.

## 4. Discussion

The primary aim of this study was to describe the prevalence of ATN profiles with definitions using exclusively neuroimaging data, in study populations recruited from two different centers that used center-specific acquisition protocols. Despite the two cohorts having faced different inclusion criteria and scanning protocols, and presenting with unequal diagnostic stages, distribution of A, T, and N biomarkers was comparable across cohorts within each profile. Furthermore, the ATN profile distribution of each cohort matched the expected distribution for each center population, respectively. Most of the identified profiles fit in the AD continuum in Geneva while in Zurich, most of the subjects were found to present normal biomarkers.

The Geneva and Zurich cohorts included subjects with significantly different clinical profiles, with the first consisting of subjects with cognitive complaints referred to a primary memory clinic, and the second consisting of healthy volunteers and subjects at an initial stage of cognitive decline that participated in a population study. Significant differences were found in years of education and MMSE scores between cohorts, which might have affected biomarker profiles. Previous studies have shown that subjects with a higher level of education might tolerate a higher level of amyloid pathology without showing clinical symptoms (50), for example. While the Geneva cohort is a sample of subjects recruited directly from a memory clinic, the Zurich cohort is comparable to a research cohort that is mostly composed of volunteers. It is known that this type of subject recruitment may lead to self-selection bias, where subjects usually have a higher degree of education, which is related to a better cognitive reserve (51). Therefore, significant differences could be expected to be found between cohorts. In fact, these differences were observed in PET measurements of centiloid and global tau in the complete cohorts, however corrected when stratified by ATN profile.

In this study, subjects were classified based on cognitive status. Resulting groups were thus comprised of individuals with different clinical diagnoses. While the Geneva CU cohort was composed of healthy volunteers as well as subjects with subjective cognitive decline, the Zurich CU cohort contained only healthy subjects.

Considering all this, our initial hypothesis that both cohorts would have different distributions of ATN profiles was well funded, graphically represented in Figure 2. Yet, both cohorts presented a normal biomarker profile as the most prevalent, with 29 and 64% of all subjects classified as A−T−N− in, respectively, Geneva and Zurich. These profiles distributions are consistent with what was observed in previous studies using PET and CSF data combined (11, 19, 20). However, it is important to notice that, in the case of this study, the use of neuroimaging biomarkers alone yielded these consistent results, with no need of harmonizing data from the two different centers. While the centiloid scale provides a harmonized measurement between the different A radiotracers that were used, no other *a priori* harmonization approach was used. This aspect was carefully chosen to better replicate conditions that are seen in clinical practice, where each centers has specific scanners, scanning protocols, and image reconstruction algorithms.

Consistent with what would be expected in a healthy population, the A−T−N− profile was mostly represented by CU individuals in the Zurich cohort. However, the same profile was composed of 54% of CI subjects in Geneva. This shows the heterogeneity and different characteristics of the recruited population in Geneva. Yet, as expected, AD continuum profiles were mostly composed of CI individuals, further demonstrating the relevance of stratifying patients based on their pathological profile, as CI subjects may now be further investigated as possible AD patients and might benefit from different care and preventive measures as compared to subjects with other diagnoses.

This study has important implications especially for the selection of subjects for clinical trials. Most studies in the field of ATN profiling are performed using cohorts selected specifically for research and, therefore, suffer from self-selection bias and may not represent the general population. The individuals from the Geneva cohort used in this study were recruited from a memory clinic and were representative of a typical population of this kind of clinic. The main strength of this study lies in the fact that it compares the prevalence of ATN profiles of patients from a memory clinic with a population that resembles more closely a research cohort (Zurich individuals).

It is important to point out that the small number of subjects in some of the profiles prevented this study from having enough statistical power to reach significant conclusions in those profiles. This was mainly the case in the SNAP profiles, which are not common within the ATN framework. However, this was also the case for the A + T + N− and A + T + N+ profiles in the Zurich cohort as it was mainly comprised of subjects at an early stage of the AD spectrum. Another limitation of this study is the long timeframe that was defined as a maximum time between assessment. However, 12 months is a usually acceptable timeframe for neurodegenerative conditions that do not progress rapidly, and no significant differences that could impact these results of this study were found.

This study aimed at characterizing two different populations in relation to ATN profiles using neuroimaging data alone. However, much remains to be explored about the two assessed populations and their classifications, such as the relationship of ATN profiles with the presence of the apolipoprotein E4 gene and other tests of clinical performance other than the MMSE scores. Furthermore, although PET provides excellent estimates for A and T biomarkers, it is an expensive technique that is not available in all hospitals. Therefore, a study exploring the correlation between ATN profiles using neuroimaging data versus CSF and blood biomarkers is still necessary. Furthermore, a study which standardizes imaging acquisition protocols between cohorts could be of interest to assess whether differences between the cohorts could be further reduced.

Neuroimaging provides solid biomarkers for ATN profiling, despite differences in subject inclusion criteria and imaging protocols used, allowing to select similar populations out of different cohorts, despite the absence of harmonization between different centers. Furthermore, the biomarker measures of A, T, and N were comparable between profiles across cohorts. The reliability of the ATN profiling in a clinical cohort represents an important step in the perspective of its use in clinical practice.

## Data availability statement

The raw data supporting the conclusions of this article will be made available by the authors, without undue reservation.

## Ethics statement

The studies involving human participants were reviewed and approved by the Commission cantonale d’éthique de la recherche— CCER de Genève and the local ethics review board in Zurich. All patients/participants provided their written informed consent to participate, and the studies were conducted in concordance with the principles of the Declaration of Helsinki and the International Conference on Harmonization Good Clinical Practice.

## Author contributions

DP contributed to the study design, image processing, data analysis, and writing and revision of the manuscript. FR contributed to the study design, image processing, and revision of the manuscript. MS contributed to subject inclusion, data acquisition, and revision of the manuscript. LM contributed to data acquisition and revision of the manuscript. VT, AG, and CH contributed to the study design, coordination of the study in Zurich, subject inclusion, data acquisition, and revision of the manuscript. GF and VG contributed to the study design, coordination of the study in Geneva, patient inclusion, data acquisition, and revision of the manuscript. All authors read and approved the manuscript.

## Funding

The Centre de la mémoire was funded by the following private donors under the supervision of the Private Foundation of Geneva University Hospitals: A.P.R.A.—Association Suisse pour la Recherche sur la Maladie d’Alzheimer, Genève; Fondation Segré, Genève; Race Against Dementia Foundation, London, UK; Fondation Child Care, Genève; Fondation Edmond J. Safra, Genève; Fondation Minkoff, Genève; Fondazione Agusta, Lugano; McCall Macbain Foundation, Canada; Nicole et René Keller, Genève; Fondation AETAS, Genève. Competitive research projects have been funded by: H2020 (projects n. 667375), Innovative Medicines Initiative (IMI contract nos. 115736 and 115952), IMI2, Swiss National Science Foundation (projects nos. 320030_182772 and 320030_169876), VELUX Foundation. VG was supported by the Swiss National Science Foundation (projects nos. 320030_169876, 320030_185028, and IZSEZ0_188355), by the Velux foundation (project no. 1123), the Schmidheiny foundation, and the Aetas foundation. Data from the Zurich cohort was supported by the Mäxi Foundation, GE HealthCare (#B023), institutional support from the institute for Regenerative Medicine, University of Zurich, Switzerland. Open access funding by the University of Geneva.

## Conflict of interest

The authors declare that the research was conducted in the absence of any commercial or financial relationships that could be construed as a potential conflict of interest.

## Publisher’s note

All claims expressed in this article are solely those of the authors and do not necessarily represent those of their affiliated organizations, or those of the publisher, the editors and the reviewers. Any product that may be evaluated in this article, or claim that may be made by its manufacturer, is not guaranteed or endorsed by the publisher.
