# Supplementary material for: ATN profile classification across two independent prospective cohorts
Source: Front Med (Lausanne). 2023 Jul 25;10:1168470. doi: 10.3389/fmed.2023.1168470 (PMC10407659; doi:10.3389/fmed.2023.1168470)
Supplement: Supplementary file 1 [file Table_1.docx]

**Supplementary Table 1.** *Descriptive features of the included cohorts by ATN profile.* Demographic, cognitive, and imaging characteristics of subjects included in the study by ATN profile and cohort. Abbreviations: y = years, F = female, M = male, MMSE = Mini-Mental State Examination, A = amyloid, T = tau, N = neurodegeneration, SUVR = standardized uptake value ratio, *Ratio between hippocampal volume and total intracranial volume

|  | **Cohort** | **A-T-N-** | **A+T-N-** | **A+T+N-** | **A+T+N+** | **A-T+N-** | **A-T-N+** | **A-T+N+** | **A+T-N+** |
| --- | --- | --- | --- | --- | --- | --- | --- | --- | --- |
| **Number of Subjects** | Geneva | 50 | 30 | 43 | 21 | 5 | 9 | 0 | 14 |
|  | Zurich | 74 | 11 | 1 | 2 | 5 | 14 | 2 | 4 |
| **Age (y)** | Geneva | 70 ± 7 | 74 ± 8 | 71 ± 9 | 76 ± 5 | 72 ± 6 | 74 ± 5 | - | 78 ± 6 |
|  | Zurich | 69 ± 8 | 71 ± 7 | 70 ± 0 | 72 ± 4 | 76 ± 5 | 76 ± 7 | 94 ± 2 | 85 ± 7 |
| **Gender (F/M)** | Geneva | 23/27 | 10/20 | 27/16 | 12/9 | 5/0 | 4/5 | - | 5/9 |
|  | Zurich | 32/42 | 1/10 | 0/1 | 0/2 | 3/2 | 2/12 | 1/1 | 0/4 |
| **Years of Education (y)** | Geneva | 15 ± 4 | 14 ± 4 | 13 ± 4 | 13 ± 4 | 12 ± 2 | 15 ± 4 | - | 13 ± 4 |
|  | Zurich | 16 ± 3 | 17 ± 3 | 20 ± 0 | 19 ± 2 | 14 ± 2 | 16 ± 3 | 17 ± 1 | 15 ± 4 |
| **MMSE** | Geneva | 28 ± 2 | 28 ± 2 | 24 ± 5 | 23 ± 4 | 28 ± 1 | 25 ± 5 | - | 25 ± 3 |
|  | Zurich | 29 ± 1 | 29 ± 1 | 28 ± 0 | 28 ± 0 | 28 ± 2 | 29 ± 1 | 29 ± 1 | 27 ± 3 |
| **Centiloid (A)** | Geneva | -3.8 ± 8.9 | 56.7 ± 37.7 | 90.1 ± 29.7 | 76.8 ± 32.8 | -7.5 ± 10.0 | -6.0 ± 7.6 | - | 66.7 ± 31.5 |
|  | Zurich | -0.8 ± 5.7 | 32.3 ± 22.2 | 19.3 ± 0 | 26.6 ± 1.5 | 2.9 ± 3.3 | -0.3 ± 8.3 | 1.8 ± 12.8 | 49.0 ± 33.4 |
| **Global Tau SUVR (T)** | Geneva | 1.11 ± 0.09 | 1.19 ± 0.14 | 1.67 ± 0.29 | 1.65 ± 0.24 | 1.33 ± 0.08 | 1.13 ± 0.11 | - | 1.22 ± 0.11 |
|  | Zurich | 1.09 ± 0.09 | 1.09 ± 0.09 | 1.82 ± 0.00 | 1.27 ± 0.01 | 1.37 ± 0.15 | 1.13 ± 0.10 | 1.30 ± 0.13 | 1.18 ± 0.08 |
| **Hippocampal Ratio***  **(×10^-3^) (N)** | Geneva | 2.6 ± 0.2 | 2.5 ± 0.2 | 2.4 ± 0.2 | 1.9 ± 0.2 | 2.5 ± 0.2 | 2.0 ± 0.1 | - | 1.9 ± 0.3 |
|  | Zurich | 2.5 ± 0.2 | 2.4 ± 0.2 | 2.2 ± 0.0 | 2.1 ± 0.1 | 2.4 ± 0.2 | 1.9 ± 0.2 | 2.0 ± 0.2 | 2.0 ± 0.1 |

**Supplementary Table 2.** *Imaging values of the cohorts by ATN profile and cognitive status.* Centiloid, global tau SUVR, and hippocampal ratio average and standard deviations by ATN profile and cognitive status for the Geneva and Zurich cohorts. Abbreviations: A = amyloid, T = tau, N = neurodegeneration, n = number of subjects, CU = cognitively unimpaired, CI = cognitively impaired, *Ratio between hippocampal volume and total intracranial volume.

| **Profile** | **Cognitive Status**  **(n Geneva/ n Zurich)** | **Centiloid** | | **Global tau SUVR** | | **Hippocampal Ratio***  **(× 10^-3^)** | |
| --- | --- | --- | --- | --- | --- | --- | --- |
|  |  | **Geneva** | **Zurich** | **Geneva** | **Zurich** | **Geneva** | **Zurich** |
| **A-T-N-** | **CU (27/60)** | -3 ± 7 | -1 ± 5 | 1.1 ± 0.1 | 1.1 ± 0.1 | 2.5 ± 0.2 | 2.5 ± 0.2 |
|  | **CI (23/14)** | -5 ± 11 | -1 ± 7 | 1.1 ± 0.1 | 1.1 ± 0.1 | 2.6 ± 0.3 | 2.5 ± 0.2 |
| **A+T-N-** | **CU (9/9)** | 51 ± 37 | 25 ± 11 | 1.1 ± 0.2 | 1.1 ± 0.1 | 2.5 ± 0.2 | 2.4 ± 0.1 |
|  | **CI (21/2)** | 59 ± 39 | 66 ± 33 | 1.2 ± 0.1 | 1.1 ± 0.1 | 2.4 ± 0.2 | 2.2 ± 0.0 |
| **A+T+N-** | **CU (1/1)** | 116 ± 0 | 19 ± 0 | 1.4 ± 0.0 | 1.8 ± 0.0 | 2.4 ± 0.0 | 2.2 ± 0.0 |
|  | **CI (42/0)** | 89 ± 30 | - | 1.7 ± 0.3 | - | 2.4 ± 0.3 | - |
| **A+T+N+** | **CU (0/0)** | - | - | - | - | - | - |
|  | **CI (21/2)** | 77 ± 32 | 27 ± 1 | 1.6 ± 0.2 | 1.3 ± 0.0 | 1.9 ± 0.2 | 2.1 ± 0.0 |
| **A-T+N-** | **CU (4/2)** | -5 ± 10 | 3 ± 5 | 1.3 ± 0.1 | 1.3 ± 0.0 | 2.5 ± 0.2 | 2.6 ± 0.1 |
|  | **CI (1/3)** | -16 ± 0 | 3 ± 3 | 1.3 ± 0.0 | 1.4 ± 0.2 | 2.2 ± 0.0 | 2.4 ± 0.2 |
| **A-T-N+** | **CU (1/8)** | -6 ± 0 | 0 ± 6 | 1.2 ± 0.0 | 1.1 ± 0.1 | 1.8 ± 0.0 | 1.9 ± 0.2 |
|  | **CI (8/6)** | -6 ± 8 | -1 ± 11 | 1.1 ± 0.1 | 1.2 ± 0.1 | 2.1 ± 0.1 | 2.0 ± 0.2 |
| **A-T+N+** | **CU (0/1)** | - | 11 ± 0 | . | 1.4 ± 0.0 | - | 1.9 ± 0.0 |
|  | **CI (0/1)** | - | -7 ± 0 | - | 1.2 ± 0.0 | - | 2.1 ± 0.0 |
| **A+T-N+** | **CU (0/1)** | - | 14 ± 0 | - | 1.1 ± 0.0 | - | 2.1 ± 0.0 |
|  | **CI (14/3)** | 67 ± 31 | 61 ± 29 | 1.2 ± 0.1 | 1.2 ± 0.1 | 1.9 ± 0.3 | 2.0 ± 0.0 |
